# The taxonomy of *Enterobacter sakazakii*: proposal of a new genus *Cronobacter *gen. nov. and descriptions of *Cronobacter sakazakii *comb. nov. *Cronobacter sakazakii *subsp. *sakazakii*, comb. nov., *Cronobacter sakazakii *subsp. *malonaticus *subsp. nov., *Cronobacter turicensis *sp. nov., *Cronobacter muytjensii *sp. nov., *Cronobacter dublinensis *sp. nov. and *Cronobacter *genomospecies 1

**DOI:** 10.1186/1471-2148-7-64

**Published:** 2007-04-17

**Authors:** Carol Iversen, Angelika Lehner, Niall Mullane, Eva Bidlas, Ilse Cleenwerck, John Marugg, Séamus Fanning, Roger Stephan, Han Joosten

**Affiliations:** 1 Quality and Safety Department, Nestlé Research Centre, Vers-Chez-les-Blanc, CH-1000 Lausanne, Switzerland; 2 Institute for Food Safety and Hygiene, Vetsuisse Faculty, University of Zurich, CH-8057, Zurich, Switzerland; 3 Centre for Food Safety, UCD Veterinary Sciences Centre, University College Dublin, Belfield, Dublin 4, Ireland; 4 BCCM/LMG Bacteria Collection, Laboratorium voor Microbiologie – Universiteit Gent, K.L. Ledeganckstraat 35, B-9000 Gent, Belgium

## Abstract

**Background:**

*Enterobacter sakazakii *is an opportunistic pathogen that can cause infections such as necrotizing enterocolitis, bacteraemia, meningitis and brain abscess/lesions. When the species was defined in 1980, 15 biogroups were described and it was suggested that these could represent multiple species. In this study the taxonomic relationship of strains described as *E. sakazakii *was further investigated.

**Results:**

Strains identified as *E. sakazakii *were divided into separate groups on the basis of f-AFLP fingerprints, ribopatterns and full-length 16S rRNA gene sequences. DNA-DNA hybridizations revealed five genomospecies. The phenotypic profiles of the genomospecies were determined and biochemical markers identified.

**Conclusion:**

This study clarifies the taxonomy of *E. sakazakii *and proposes a reclassification of these organisms.

## Background

*Enterobacter sakazakii *was defined as a new species in 1980 by Farmer *et al *[1]. DNA-DNA hybridization gave no clear generic assignment for *E. sakazakii *as it was shown to be 53–54% related to species in two different genera, *Enterobacter *and *Citrobacter*. However the species was placed in *Enterobacter *as it appeared phenotypically and genotypically closer to *E. cloacae *than to *C. freundii*, the type species of these genera. In the original study fifteen biogroups of *E. sakazakii *were described. Recently the existence of a sixteenth biogroup has been reported and a correlation between 16S rRNA gene sequence analysis, which separated *E. sakazakii *strains into several genetic groups, and biogroups has been demonstrated [2-4]. The existence of these divergent groups seems to support the suggestion of Farmer *et al *that *E. sakazakii *may harbour different species [1]. However previous studies were based on only partial (~500 bp) 16S rRNA gene sequence analysis, whereas for taxonomical purposes the complete gene should be sequenced (>1300 bp with less than 0.5% undetermined bases) [5,6]. It has until recently been generally accepted that it is unlikely that two bacterial strains belong to the same species if the similarity between their 16S rRNA genes is <97% [7-9], but based on an extensive evaluation of published data this value has been amended to a range between 98.7–99%. [10]. For strains whose 16S rRNA gene similarity exceeds this threshold value, a DNA-DNA reassociation assay must be performed and only if this test reveals more than 70% relatedness can it be concluded that the strains belong to the same species [7,11-14].

DNA profiling methods such as ribotyping [15,16] and amplified fragment length polymorphisms (AFLP) have been shown to discriminate at the species and subspecies level and may provide valuable additional information in taxonomic studies. The AFLP technique has been employed in plant and microbiological research to describe the molecular ecology of various niches and this technique can be used to determine inter- and intra-species relatedness [17-19]. Mougel *et al *[20] found that members of the same genomic species cluster consistently using AFLP analysis and suggested that future genomic delineation of bacterial species could be based on this approach.

In this study, independent molecular methods, including f-AFLP, automated ribotyping, full-length 16S rRNA gene sequencing and DNA-DNA hybridization, were employed to clarify the taxonomic relationship of 210 strains currently described as *E. sakazakii *and amendments to the classification of these organisms are proposed.

## Results and Discussion

**Figure 1:**
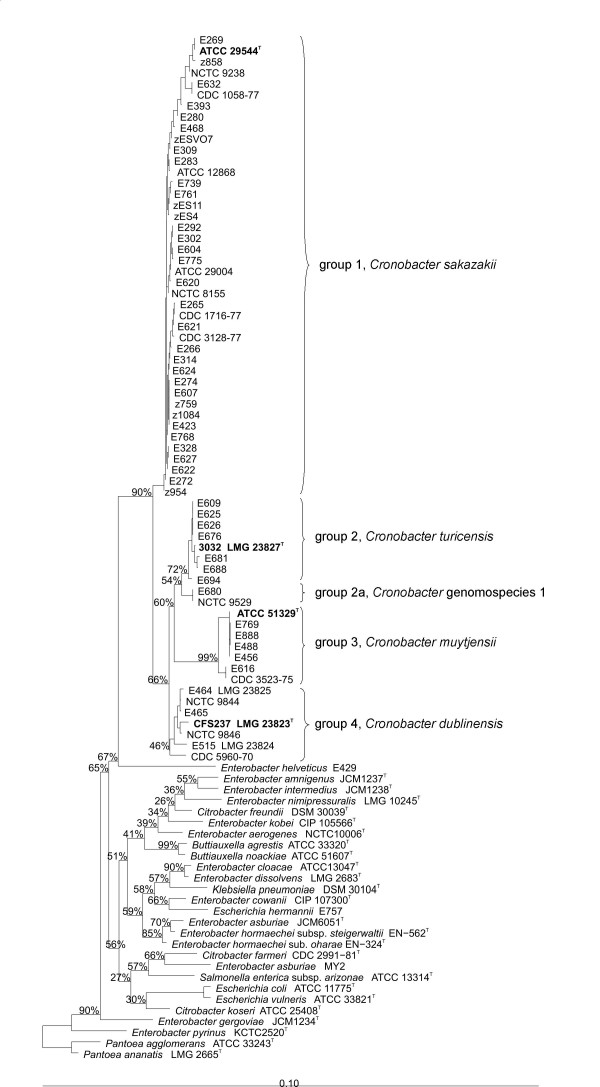
**16S rRNA gene phylogenetic tree of *Cronobacter *and related species**. A Neighbor-Joining analysis was used with Felsenstein correction (1000 bootstrap replicates). The bar indicates 10% estimated sequence divergence.

**Figure 2:**
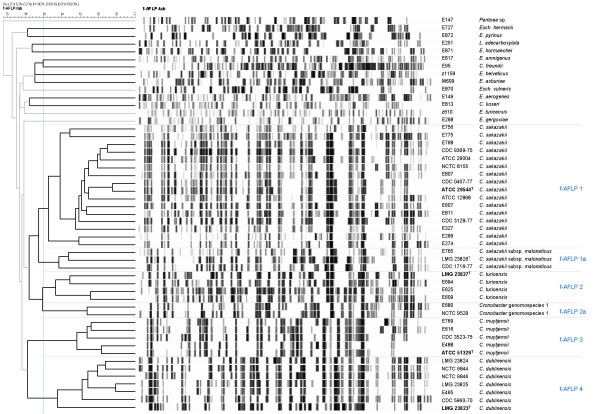
**f-AFLP dendogram of *Cronobacter *and related species**. A DICE coefficient and UPGMA algorithm were used with an optimization of 0% and position tolerance of 0.2%. The scale bar represents the percentage of similarity.

**Figure 3:**
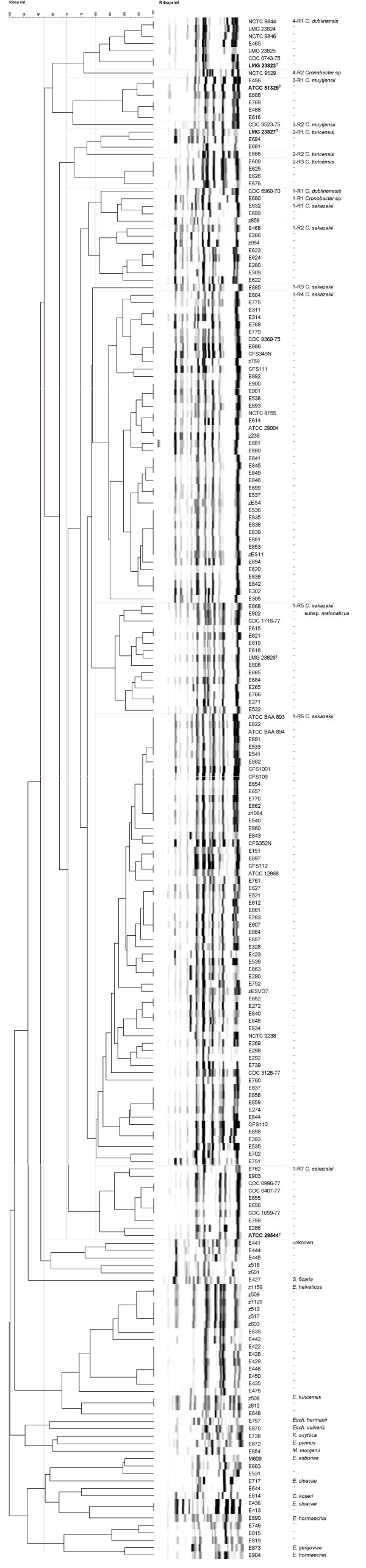
**Ribotype dendogram of *Cronobacter *and related species**. A DICE coefficient and UPGMA algorithm were used with an optimization of 1% and position tolerance of 1.5%. The scale bar represents the percentage of similarity.

The 210 *E. sakazakii *strains were assigned to biogroups as originally described by Farmer *et al *[1] with the addition of biogroup 16 as described by Iversen *et al *[4]. The defining characteristics used to identify each biogroup (Additional file 1) are as previously described [1,4]. Full length 16S rRNA gene sequences, comprising greater than 1300 bp with less than 0.5% undetermined positions, were obtained for 66 *E. sakazakii *strains representative of the different biogroups and 13 strains representative of other species. Additional sequences were downloaded from the EMBL database. In agreement with previous partial 16S rRNA gene sequencing, the majority of the full-length *E. sakazakii *16S rRNA gene sequences clustered closely with the type strain, *E. sakazakii *ATCC 29544^T ^(Figure 1 – group 1). The remaining sequences formed three clusters (Figure 1 – groups 2–4). Representative strains (n = 51) were compared using f-AFLP (Figure 2). Calculation of point-bisectional correlations to statistically delimit relevant clusters resulted in two groups equating to 16S rRNA groups 1 & 2 and 16S rRNA groups 3 & 4. At a cut off level of 50% similarity six clusters could be delineated, which corresponded to strains belonging to 16S rRNA groups 1 to 4 plus a subset of group 1 comprising strains of biogroups 5 & 9 (Figure 2 – f-AFLP group 1a) and a subset of group 2 (Figure 2 – f-AFLP group 2a). Ribotyping was performed for 209 strains including representatives of all different biogroups (Figure 3). All the *E. sakazakii *strains shared more than 62% pattern similarity whereas other *Enterobacteriaceae *shared less than 62% pattern similarity with the *E. sakazakii *strains. A similarity of greater than 70% was used to delineate separate *E. sakazakii *groups resulting in four clusters 1-R, 2-R, 3-R, and 4-R, which correspond to 16S rRNA groups 1–4.

DNA-DNA hybridizations were performed with two strains from each of the four groups (Table 1). The *E. sakazakii *type strain, ATCC 29544^T^, and ATCC 12868 (group 1) showed 70% DNA relatedness suggesting that they belong to the same species considering that a DNA homology of 70% is generally regarded as the limit for species delineation [14]. Although the value appears low, the similarity between the 16S rRNA gene sequences for these two strains was 99.6% and they clustered closely using the f-AFLP analysis and ribotyping, which supports their same species relationship. Previously, DNA-DNA hybridization of 13 strains of *E. sakazakii *resulted in relative binding ratios between 72–95% at an optimal renaturation temperature of 59.5°C [21]. Group 1 comprises biogroups 1–5, 7–9, 11, 13, and 14 as described by Farmer *et al *[1]. Isolates belonging to biogroups 5, 9 and 14 grouped together as a subcluster of group 1 in the ribotype analysis (Figure 3 – 1-R5) and representative strains of this subgroup also formed a coherent cluster in the f-AFLP analysis (Figure 2 – f-AFLP 1a). No other relationship between biogroup and subcluster was identified for the other strains within group 1. The 16S rRNA gene sequences of biogroup 5, 9 and 14 strains displayed 99.6% similarity with ATCC 29544^T^. These strains share the common feature of being positive for malonate utilization and are proposed as a subspecies of group 1.

**Table 1:** DNA-DNA % relatedness of *Cronobacter *strains (in 2 × SSC + 5% formamide at 70°C)

	*C. sakazakii *ATCC 29544^T^	*C. sakazakii *ATCC 12868	*C. turicensis *3032 (LMG 23827^T^)	*C. muytjensii *ATCC 51329^T^	*C. dublinensis *NCTC 9844
*C. sakazakii *ATCC 12868	70.0 (2.4)^§^				
*C. turicensis *3032 (LMG 23827^T^)	51.8 (2.8)	51.9 (2.6)			
*C. muytjensii *ATCC 51329^T^	53.3 (3.0)	42.0 (4.0)	56.0 (4.2)		
*C. muytjensii *CDC 3523-75	37.7 (1.8)	31.3 (6.2)		91.9 (4.9)	
*C. dublinensis *NCTC 9844	16.7 (7.6)	43.0 (9.8)	36.8 (2.2)	54.5 (4.7)	
*C. dublinensis *CDC 5960-70	37.3 (0.0)	54.8 (2.1)			77.4 (7.5)
*Cronobacter *genomospecies 1 NCTC 9529	55.5 (1.0)	54.4 (3.9)	55.0 (3.3)	53.1 (6.6)	45.9 (2.0)

^§^% relatedness values are averages of duplicate measurements and standard deviations are given in parentheses (values can be reproduced in a range of about 10%).

Two strains from different branches within group 3, ATCC 51329^T ^and CDC 3523-75, showed 92% DNA relatedness whereas their relatedness to the group 1 strains was only 31–53% (Table 1). Also the similarity of the 16S rRNA gene between group 3 strains was 99.9% but only 97.9% similarity was shared with ATCC 29544^T ^(Table 2). This is below the threshold of 98.7% recently proposed at which DNA-DNA hybridization is mandatory [10]. Further, these strains shared greater than 50% pattern similarity using f-AFLP analysis but less than 50% similarity to all other strains (Figure 2). From these results it can be concluded that the group 3 strains represent a single distinct species from ATCC 29544^T^. Group 3 comprises strains identified as biogroup 15 [1].

**Table 2:** 16S rRNA gene % similarity of *Cronobacter *species type strains to *Cronobacter *species and related *Enterobacteriaceae*.

		*C. sakazakii *ATCC 29544^T^	*C. turicensis *3032 LMG 23827^T^	*C. dublinensis *CFS237 LMG 23823^T^	*C. muytjensii *ATCC 51329^T^	*Cronobacter *genomospecies 1 NCTC 9529
*C. sakazakii *sp.	(44)^§^	99.6 ± 0.23^2^	98.9 ± 0.13	98.9 ± 0.13	98.3 ± 0.19	98.9 ± 0.18
*C. turicensis *sp.	(8)	98.7 ± 0.08	100.0 ± 0.04	99.5 ± 0.07	98.4 ± 0.07	99.6 ± 0.07
*C. dublinensis *sp.	(7)	98.7 ± 0.19	99.4 ± 0.14	99.6 ± 0.24	98.4 ± 0.17	99.2 ± 0.17
*C. muytjensii *sp.	(7)	97.9 ± 0.00	98.4 ± 0.13	98.3 ± 0.15	99.9 ± 0.20	98.7 ± 0.10
*Cronobacter *genomospecies 1	(2)	98.5 ± 0.00	99.6 ± 0.00	99.1 ± 0.00	98.6 ± 0.00	100.0 ± 0.00
*Citrobacter *spp.	(5)	96.8 ± 0.46	96.5 ± 0.33	96.5 ± 0.32	97.1 ± 0.44	96.7 ± 0.34
*Enterobacter *spp.	(24)	96.8 ± 0.04	96.6 ± 0.16	96.4 ± 0.08	97.2 ± 0.12	96.7 ± 0.12

^§^number of full-length 16S rRNA gene sequences compared. ^2^mean % similarity of the sequences to the *Cronobacter *species type strains ± standard deviation.

Group 4 is comprised of strains identified as biogroups 6, 10 and 12 along with NCTC 9846. DNA-DNA hybridization of CDC 5960-70 (biogroup 6) and NCTC 9844 (biogroup 10) showed 77% DNA relatedness, whereas the DNA relatedness to the group 1 strains was 16–55% (Table 1). Also the 16S rRNA gene similarity within group 4 was 99.6% compared with a similarity to ATCC 29544^T ^of 98.7% (Table 2). Using f-AFLP analysis group 4 strains shared more than 50% pattern similarity supporting the conclusion that these all belong to the same novel species (Figure 2). This leads to the conclusion that the group 4 strains represent a single distinct species from ATCC 29544^T^.

Group 2 equates to strains recently identified as biogroup 16. Two strains from different branches within this group, 3032 (= LMG 23827^T^) and NCTC 9529, showed approximately 55% DNA homology, which is clearly below 70%, suggesting the two branches within group 2 represent different species. The DNA relatedness of LMG 23827^T ^to group 1 was approximately 52% and the 16S rRNA gene similarity was 98.7%. The DNA relatedness of NCTC 9529 to group 1 was approximately 55% with 98.5% 16S rRNA gene similarity (Tables 1 &2). The 16S rRNA gene sequences for all strains in group 2 were more than 99% similar to each other, but were only 98.5–98.7% similar to the 16S rRNA gene sequence of *E. sakazakii *ATCC 29544^T ^(Table 2). The ribotype analysis clustered eight of the group 2 strains together with a pattern similarity of greater than 70% (Figure 3). These strains also shared more than 50% pattern similarity in the f-AFLP analysis (Figure 2). However two strains, NCTC 9529 and E680, did not cluster with the other biogroup 16 strains using ribotyping. Also, although they consistently clustered in close proximity, both strains shared less than 50% pattern similarity to the other biogroup 16 strains based on f-AFLP analysis (Figure 2). This indicates there are two species within group 2 and both of these are distinct species from ATCC 29544^T^.

DNA homology values amongst strains belonging to different groups were all clearly below 70%, therefore it was concluded that the *E. sakazakii *strains in this study represent five separate species (Table 1). Statistical analysis of phenotypic profiles (using a Fisher's exact test) showed that important biochemical tests for species differentiation were indole production, malonate utilization and acid production from dulcitol and methyl-α-D-glucoside (Table 3). Phenotypic profiles allowed the differentiation of four DNA hybridization groups and within group 1 two phenotypic subgroups could be delineated. NCTC 9529 and E680 could not be distinguished from the other biogroup 16 strains using the tests within this study.

**Table 3:** Statistically relevant biochemical tests for the differentiation of proposed *Cronobacter *species and subspecies.

		Dul	Ind	Malo	AMG
*Cronobacter sakazakii *subsp. *sakazakii*	(163)^§^	-	-	-	+
*Cronobacter sakazakii *subsp. *malonaticus*	(22)	-	-	+	+
*Cronobacter muytjensii*	(7)	+	+	+	-
*Cronobacter dublinensis*	(8)	-	+	v	+
*Cronobacter turicensis*	(8)	+	-	+	+
*Cronobacter *genomospecies 1	(2)	+	-	+	+

^§^number of isolates; Dul, production of acid from dulcitol; Ind, production of indole; Malo, malonate utilization; AMG, production of acid from methyl-α-D-glucoside; +, 85–100% positive; v, 15–85% positive; -, less than 15% positive.

It is important, however, that reclassification of species is not detrimental to health protection measures already in place and that all risk organisms continue to be recognized. The different species currently identified as *E. sakazakii *contain clinical isolates cultured from body sites that would normally be sterile. As there is insufficient evidence regarding the virulence potential of these species to conclude that any one of them does not represent a health risk to neonates, it is proposed that *Enterobacter sakazakii *be reclassified as four species, one genomospecies, and two subspecies in a new genus within the family *Enterobacteriaceae*. These organisms are a microbiological hazard occurring in the infant food chain with historic high morbidity and mortality in neonates [22]. Therefore *Cronobacter *gen. nov. is proposed after the Greek mythological god, Cronos, who was described as swallowing his children at birth [23]. This genus would contain the type species, *C. sakazakii *comb. nov. (comprising group 1 strains) with *C. sakazakii *subsp. *sakazakii *comb. nov. and *C. sakazakii *subsp. *malonaticus *subsp. nov.; *C. muytjensii *sp. nov. (group 3 strains); *C. dublinensis *sp. nov. (group 4 strains); *C. turicensis *sp. nov. (the majority of group 2 strains); and, as NCTC 9529 and E680 cannot be phenotypically distinguished from *C. turicensis *subsp. nov. and there are only two of these strains, it is proposed that they are referred to as *Cronobacter *genomospecies 1 for the present. The phenotypic differentiation of *Cronobacter *spp. from other common *Enterobacteriaceae *genera is presented in Table 4.

**Table 4:** Biochemical differentiation of *Cronobacter *from other *Enterobacteriaceae*.

	4-NP-α-Glc	VP	ADH	ODC	SAC	RAF	CEL	ARA	CIT	MR	ADO	SOR	LDC	H_2_S
*Cronobacter *spp.	+	+	+	+	+	+	+	+	+	-	-	-	-	-
*Buttiauxella agrestis*	v	-	-	+	-	+	+	+	+	+	-	-	-	-
*Citrobacter koseri*	-	-	v	+	v	-	+	+	+	+	+	+	-	-
*Citrobacter freundii*	-	-	v	-	v	v	v	+	v	+	-	+	-	+
*Edwardsiella tarda*	-	-	-	+	-	-	-	-	-	+	-	-	+	+
*Enterobacter aerogenes*	-	+	-	+	+	+	+	+	+	-	+	+	+	-
*Enterobacter asburiae*	-	-	v	+	+	v	+	+	+	+	-	+	-	-
*Enterobacter cancerogenus*	-	+	+	+	-	-	+	+	+	-	-	-	-	-
*Enterobacter cloacae*	-	+	+	+	+	+	+	+	+	-	v	+	-	-
*Enterobacter gergoviae*	-	+	-	+	+	+	+	+	+	-	-	-	+	-
*Enterobacter hormaechei*	-	+	v	+	+	-	+	+	+	v	-	-	-	-
*Enterobacter pyrinus*	v	v	-	+	+	-	+	+	-	v	-	-	+	-
Enterobacter helveticus*	+	-	-	-	-	-	+	+	-	+	-	-	-	-
Enterobacter turicensis*	+	-	-	-	-	-	+	+	-	+	-	-	-	-
*Escherichia coli*	-	-	v	v	v	v	-	+	-	+	-	+	(+)	-
*Hafnia alvei*	(-)	(+)	-	+	-	-	(-)	+	-	v	-	-	+	-
*Klebsiella pneumoniae*	(-)	+	-	-	+	+	+	+	+	-	+	+	+	-
*Kluyvera *spp.	v	-	-	+	+	+	+	+	(+)	+	-	v	v	-
*Leclercia adecarboxylata*	-	-	-	-	v	v	+	+	-	+	+	-	-	-
*Morganella morganii*	-	-	-	+	-	-	-	-	-	+	-	-	-	(-)
*Pantoea *spp.	-	v	-	-	v	v	v	+	v	v	-	v	-	-
*Proteus vulgaris*	+	-	-	v	(+)	-	-	-	v	v	-	-	-	+
*Providencia *spp.	-	-	-	-	v	-	-	-	v	+	v	-	-	v
*Rahnella aquatilis*	-	-	v	-	+	+	+	+	(-)	-	-	+	-	-
*Raoultella terrigena*	-	+	-	(-)	+	+	+	+	v	v	+	+	+	-
Salmonella sv.	-	-	v	(+)	-	-	v	(+)	v	+	-	v	(+)	v
*Serratia marcescens*	v	+	-	+	+	-	-	-	+	(-)	v	+	+	-
*Yersinia enterocolitica*	-	-	-	+	+	-	v	+	-	+	-	+	-	-

4-NP-α-Glc, metabolism of 4-NP-α-glucoside; VP, Voges-Proskauer; ADH, arginine dihydrolase; ODC, ornithine decarboxylase; SAC, acid from sucrose; RAF, acid from raffinose; CEL, acid from cellobiose; ARA, acid from arabinose; CIT, use of citrate as sole carbon source (Simmon's); ADO, acid from adonitol; SOR, acid from sorbitol; LDC, lysine decarboxylase; MR, methly red test; H2S, production of hydrogen sulphide. +, 90–100% positive; (+), 80–90% positive; v, 20–80% positive; (-), 10–20% positive; -, less than 10% positive. Data was derived from this study and from Manual of Clinical Microbiology, 7th Edition [25]. *Enterobacter helveticus and *E. turicensis *are novel *Enterobacter *species [38].

Isolates associated with neonatal meningitis were identified as belonging to *C. sakazakii *subsp. *sakazakii *subsp. nov., *C. sakazakii *subsp. *malonaticus *subsp. nov. and *C. turicensis *sp. nov. However, *C. muytjensii *sp. nov. and *C. dublinensis *sp. nov. contained human isolates from normally sterile sites, bone marrow (CDC 3523-75) and blood (CDC 5960-70) respectively [1]. The creation of a new genus simplifies the inclusion of these potentially pathogenic organisms in legislation and current identification schemes developed for *E. sakazakii *remain applicable for the *Cronobacter *genus.

### Description of the genus *Cronobacter *gen.nov

The genus *Cronobacter *comprises oxidase negative, catalase positive, facultative anaerobic, peritrichous, Gram negative rods approximately 3 μm by 1 μm in size. They are generally motile, reduce nitrate, utilize citrate, hydrolyze esculin and arginine, and produce acid from D-glucose, D-sucrose, D-raffinose, D-melibiose, D-cellobiose, D-mannitol, D-mannose, L-rhamnose, L-arabinose, D-xylose, D-trehalose, galacturonate and D-maltose. *Cronobacter *strains metabolize the substrates 5-bromo-4-chloro-3-indolyl-α-D-glucopyranoside, 4-methylumbelliferyl-α-D-glucopyranoside, 4-nitophenyl-α-D-glucopyranoside, 4-nitophenyl-β-D-glucopyranoside, 4-nitophenyl-α-D-galactopyranoside and 4-nitophenyl-β-D-galactopyranoside. They are also generally positive for acetoin production (Voges-Proskauer test) and negative for the methyl red test indicating 2,3-butanediol rather than mixed acid fermentation. Negative reactions include hydrogen sulphide production, urea hydrolysis, lysine decarboxylation, β-D-glucuronidase and metabolism of D-sorbitol, erythritol, mucate, tartrate, 5-ketogluconate, D-saccharic acid, sodium pyruvate, glucose-1-phosphate, glucose-6-phosphate, adonitol and arabitol [[1,2,4,24,25] and this study]. Previously, G+C ratios of 57% and 56.7% have been reported for strains belonging to the type species [1,26].

### Description of *Cronobacter sakazakii *comb. nov. including *C. sakazakii *subsp. *sakazakii *comb. nov. and *Cronobacter sakazakii *subsp. *malonaticus *subsp. nov

*Cronobacter sakazakii *comb. nov., named in honour of the Japanese microbiologist Riichi Sakazaki when the species was first designated in 1980 as *Enterobacter sakazakii *[1], is the type species of the proposed genus *Cronobacter *and the type strain is ATCC 29544^T ^(ATCC, Manassas, VA, USA) also available as NCTC 11467^T ^(NCTC, London, UK). The type strain was originally isolated from a child's throat [1].

*C. sakazakii *subsp. *sakazakii*, comprises biogroups 1, 2, 3, 4, 7, 8, 11 and 13 previously described [1] and is generally indole, dulcitol and malonate negative, but methyl-α-D-glucopyranoside positive (Table 3).

*Cronobacter sakazakii *subsp. *malonaticus *subsp. nov. (mă.lō.nă.tĭ'cŭs. N.L. n. malonas -atis, malonate; L. suff. -icus, suffix used with the sense of belonging to; N.L. masc. adj. malonaticus, pertaining to the utilization of malonate) is comprised of biogroups 5, 9 and 14 previously described [1]. The proposed type strain for this subspecies, CDC 1058-77, was originally isolated from a breast abscess and is also available as LMG 23826^T ^(BCCM/LMG, Ghent, Belgium) and DSMZ 18702^T ^(DSMZ, Braunschweig, Germany). *C. sakazakii *subsp. *malonaticus*, is indole, and dulcitol negative, but malonate and methyl-α-D-glucopyranoside positive (Table 3).

### Description of *Cronobacter muytjensii *sp nov

*Cronobacter muytjensii *sp. nov. (mœ.tjǝn.sĭ.ī. N.L. gen. n. muytjensii, of Muytjens) named in honour of the Dutch microbiologist Harry Muytjens who performed much of the early work on *E. sakazakii *[27-31]. This species comprises biogroup 15 as previously described [1]. The proposed type strain is ATCC 51329^T ^(ATCC, Manassas, VA, USA) also available as CIP 103581^T ^(Collection de l'Institut Pasteur, Paris, France). This strain was originally deposited by bioMérieux, La Balme-les-Grottes, France. *C. muytjensii *sp. nov. is indole, dulcitol, and malonate positive but palatinose and methyl-α-D-glucopyranoside negative (Table 3).

### Description of *Cronobacter dublinensis *sp nov

*Cronobacter dublinensis *sp. nov. (dŭb.lĭn.ĕn'sĭs. N.L. masc. adj. dublinensis, pertaining to Dublin, Ireland, the origin of the type strain) is comprised of biogroups 6, 10 and 12 as previously described [1]. The type strain, CFS237, is from a milk powder manufacturing facility and is available as LMG 23823^T ^(BCCM/LMG, Ghent, Belgium) and DSMZ 18705^T ^(DSMZ, Braunschweig, Germany). *C. dublinensis *sp. nov. is dulcitol negative and methyl-α-D-glucopyranoside positive and generally positive for indole production.

### Description of *Cronobacter turicensis *sp. nov

*Cronobacter turicensis *sp. nov. (tŭ.rĭ.sĕn'sĭs. L. masc. adj. turicensis, pertaining to Turicum, the Latin name of Zurich, as the type strain originates from Zurich, Switzerland). The proposed type strain, 3032, is available as LMG 23827^T ^(BCCM/LMG, Ghent, Belgium) and DSMZ 18703^T ^(DSMZ, Braunschweig, Germany). This strain was isolated from a fatal case of neonatal meningitis occurring in Zurich in 2005 [32]. *C. turicensis *sp. nov. strains are indole negative but malonate, dulcitol and methyl-α-D-glucopyranoside positive.

### Description of *Cronobacter *genomospecies 1

As no phenotypic differentiation of these strains from other strains within biogroup 16 could be determined and only two strain exist in this group, at the present time it is proposed to designate a novel genomospecies [33] represented by strain NCTC 9529. This strain was originally isolated from water and deposited at the NCTC, London, UK, in 1954.

*Cronobacter *genomospecies 1 strains are indole negative but malonate, dulcitol and methyl-α-D-glucopyranoside positive.

## Conclusion

This study clarifies the taxonomy of *E. sakazakii *and proposes a reclassification of these organisms.

## Methods

### Sources of bacterial strains

A total of 312 strains obtained from the culture collections at Nestlé Research Centre, Lausanne, Switzerland (NRC), the Institute for Food Safety and Hygiene, Vetsuisse Faculty, University of Zurich (UZH) and the Centre for Food Safety (CFS), University College Dublin (UCD) were analyzed in this study. The strains comprised 210 isolates currently identified as *E. sakazakii *and 102 representative strains of related species. At least one strain from each of the biogroups described when the *E. sakazakii *species was designated were included [1]. The majority of strains were environmental and food isolates from NRC, UZH and UCD. Additional clinical, food and environmental isolates were originally obtained from the ATCC, Manassas, VA, USA; NCTC, London, UK; CDC, Atlanta, GA, USA; HC-SC, Health Products and Food branch, Canada; Oxoid Ltd., Basingstoke, UK; the Department of Medical Microbiology, University of Nijmegen, Netherlands; Food Safety Lab, Cornell University, Ithaca, NY, USA; US FDA Center for Food Safety and Applied Nutrition, College Park, MD, USA; R & F Laboratories, Downers Grove, IL, USA; bioMérieux, La Balme Les Grottes, France and the Institute for Medical Microbiology and Immunology, University of Bonn, Germany.

### Phenotypic characterization

Biochemical tests were performed as follows with negative tests being incubated for 7 days before discarding unless otherwise indicated. Motility was determined at 37°C using motility medium (tryptose 10 g l^-1^, NaCl 5 g l^-1^, agar 5 g l^-1^, pH 7.2 ± 0.2). Acid production from carbohydrate (D-sucrose, D-melibiose, D-raffinose, D-sorbitol, L-rhamnose, D-cellobiose, D-trehalose, palatinose, adonitol, myo-inositol, dulcitol and methyl-α-D-glucopyranoside) was tested in phenol red broth base (10 g l^-1 ^peptone, 1 g l^-1 ^yeast extract, 5 g l^-1 ^NaCl, 0.018 g l^-1 ^phenol red) with addition of filter-sterilized carbohydrate solution (final concentration 0.5%). Gas production from glucose and methyl-α-D-glucopyranoside was determined by collection in Durham tubes. Utilization of citrate as a sole carbon source was determined on Simmons Citrate agar (85463, Fluka). Malonate utilization was determined using sodium malonate broth (M8802, Sigma). The Methyl Red test was performed by addition of indicator (0.1 g methyl red per 300 ml 95% ethanol) to cultures grown for 48 h in 10 ml Methyl Red Voges-Proskauer Broth (MR-VP; 39484, Fluka). The Voges-Proskauer test was performed by addition of VP1 and VP2 reagents (bioMérieux) to cultures grown for 24 h in MR-VP broth. Indole production was measured by addition of James Reagent (70542 bioMérieux) to cultures grown for 24 h in Peptone Water (CM0009 Oxoid Ltd). Nitrate reduction was measured by addition of NIT1 and NIT2 reagents (bioMérieux) to turbid cultures in nitrate broth (72548 Fluka). Durham tubes were used for collection of nitrogen gas and zinc dust was added to negative tubes to confirm the presence of unreduced nitrate.

### DNA-DNA hybridizations

Isolates were grown in BHI 24 h at 37°C and centrifuged at 4,000 rpm for 30 min at 4°C to obtain 3 g wet biomass. Biomass was suspended in iso-propanol/H_2_O (1:1, v/v). DNA-DNA hybridizations were performed by DSMZ, Germany. DNA was isolated using a French pressure cell (Thermo Spectronic) and was purified by chromatography on hydroxyapatite as described by Cashion *et al *[34]. DNA-DNA hybridizations were carried out spectrophotometrically in 2 × SSC + 5% formamide as described by De Ley *et al *[11] at 70°C with consideration of the modifications described by Huss *et al *[13] using a model Cary 100 Bio UV/VIS-spectrophotometer equipped with a Peltier-thermostatted 6 × 6 multicell changer and a temperature controller with *in-situ *temperature probe (Varian). DNA homology values were determined in duplicate.

### AFLP

DNA was prepared using the method of Gevers *et al *[35]. Purified total DNA was digested by two restriction enzymes, a 4- and a 6-base cutter. Small DNA molecules (15–20 bp) containing one compatible end were ligated to the appropriate 'sticky end' of the restriction fragments. Restriction enzyme: *Eco*R I [hexacutter], adaptor: 5'-CTC GTA GAC TGC GTA CC-3'; 3'-CTG ACG CAT GGT TAA-5'. Restriction enzyme: *Taq *I [tetracutter], adaptor: 5'-GAC GAT GAG TCC TGA C-3'; 3'-TAC TCA GGA CTG GC-5'. Selective amplification of the restriction fragments was performed using the following primer combination: E01: 5'-GAC TGC GTA CCA ATT CA-3'; T01: 5'-CGA TGA GTC CTG ACC GAA-3'.

Amplicons were separated according to their length on a high resolution polyacrylamide gel using a DNA sequencer (ABI 377) and visualized by the 5'-end labelling of the 6-bp cutter with the fluorescent dye FAM. The resulting electrophoretic patterns were tracked and normalized using the GeneScan 3.1 software (Applera, USA). Normalized tables of peaks, containing fragments of 50 to 536 base pairs, were transferred into the BioNumerics™ 4.5 software (Applied Maths, Belgium). For numerical analysis, data intervals were delineated between the 75- and 500-bp bands of the internal size standard. Clustering of the BioNumerics™ 4.5 software generated AFLP™ DNA fingerprint patterns was performed using the Dice coefficient and the UPGMA algorithm with an optimization of 0% and position tolerance of 0.2%.

### Ribotyping

Ribotyping was performed using the automated RiboPrinter™ Microbial Characterization System (Qualicon Inc., DE, USA). Isolates were grown on TSA (18 h, 37°C) and prepared according to standard procedures [36] using the *Eco*R1 restriction enzyme. Riboprint patterns were downloaded to Bionumerics v.4.5, a UPGMA dendogram was constructed using a DICE coefficient with an optimization of 1% and a position tolerance of 1.5%.

### 16S rRNA gene sequences

16S rRNA gene sequencing was performed by Fasteris SA, Switzerland. Isolates were grown 18 h in 5 ml BHI at 37°C and 1 ml centrifuged at 13,000 g for 5 min, the pellet was washed with 1 ml H_2_O. PCR-ready DNA was prepared by washing 10 μl of the concentrated culture with 200 μl H_2_O and resuspending the pellet in 50 μl Prepman Ultra (Applied Biosystems). The cells were heated at 99°C for 10 min. Ribosomal 16S rRNA gene was amplified from 1 μl of PCR-ready DNA using the high-fidelity polymerase PrimeStar (Takara) and the primers P0 and P6 in the presence of betaine: 3 min at 95°C; 30 cycles of 30 sec at 95°C, 30 sec at 56°C, 2 min at 72°C; 5 min at 72°C. P0 – 5'-AGA GTT TGA TCC TGG CTC AG-3'; P6 – 5'-GTA CGG CTA CCT TGT TAC GA-3'. The PCR was carried out in 3 × 15 μl, which were subsequently pooled. After purification the amplified fragments were sequenced using the primers P0 and P6. Some templates produced a double sequence close to the P0 site, these were sequenced using another primer that binds in the reverse orientation to a conserved region in the middle of the 16S region (095P: 5'-TAC GGC GTG GAC TAC CAG-3'). The new reactions produced a double sequence less than 80 bases from the end of the PCR fragment (P0 site). Additional sequences were downloaded from EMBL database.

Full-length 16S rRNA gene sequences (> 1300 bases) were added to an ARB alignment of about 28,000 small-subunit rRNA sequences by using the alignment tool ARB_EDIT of the ARB program package [37]. Alignments were refined by visual inspection. The neighbor-joining method, combined with a Felsenstein correction, was used to infer the distance-matrix tree; 1,000 bootstrap replicates were performed.

### Submission of 16S rRNA gene sequences

The accession numbers of the 16S rRNA genes included in this study are as follows: ATCC 12868 [GenBank: EF059844], ATCC 29004 [GenBank: EF059868], ATCC 29544T [GenBank: EF059843], ATCC 51329 [GenBank: EF059845], CDC 1716-77 [GenBank: EF059883], CDC 3128-77 [GenBank: EF059882], CDC 3523-75 [GenBank: EF059875], CDC 5960-70 [GenBank: EF059874], E265 [GenBank: AY803191], E266 [GenBank: AY803190], E269 [GenBank: EF059819], E271 [GenBank: EF059820], E272 [GenBank: EF059821], E274 [GenBank: EF059822], E280 [GenBank: EF059823], E283 [GenBank: EF059824], E292 [GenBank: EF059825], E302 [GenBank: EF059826], E309 [GenBank: EF059827], E314 [GenBank: EF059828], E328 [GenBank: EF059829], E393 [GenBank: AY752941], E413 [GenBank: EF059830], E423 [GenBank: EF059831], E429 [GenBank: EF059832], E436 [GenBank: EF059833], E440 [GenBank: EF059834], E444 [GenBank: EF059835], E450 [GenBank: EF059836], E456 [GenBank: EF059837], E465 [GenBank: EF059839], E468 [GenBank: AY752942], E488 [GenBank: EF059840], E531 [GenBank: EF059842], E604 [GenBank: EF059846], E607 [GenBank: EF059847], E609 [GenBank: EF059848], E616 [GenBank: EF059849], E620 [GenBank: EF059850], E621 [GenBank: EF059851], E622 [GenBank: EF059852], E624 [GenBank: EF059853], E625 [GenBank: EF059854], E626 [GenBank: EF059855], E627 [GenBank: EF059856], E632 [GenBank: EF059857], E639 [GenBank: EF059858], E644 [GenBank: EF059859], E676 [GenBank: EF059860], E680 [GenBank: EF059861], E681 [GenBank: EF059862], E688 [GenBank: EF059863], E694 [GenBank: EF059864], E717 [GenBank: EF059865], E739 [GenBank: EF059867], E757 [GenBank: EF059869], E761 [GenBank: EF059870], E768 [GenBank: EF059871], E769 [GenBank: EF059872], E775 [GenBank: EF059873], E814 [GenBank: EF059880], E872 [GenBank: EF059884], E883 [GenBank: EF059886], E888 [GenBank: EF059887], E890 [GenBank: EF059888], E895 [GenBank: EF059889], E904 [GenBank: EF059890], CFS237 LMG 23823 [GenBank: EF059892], LMG 23824 [GenBank: EF059841], E464 LMG 23825 [GenBank: EF059838], CDC 1058-77 LMG 23826 [GenBank: EF059881], 3032 LMG 23827 [GenBank: EF059891], M609 [GenBank: EF059885], NCTC 8155 [GenBank: EF059866], NCTC 9238 [GenBank: EF059876], NCTC 9529 [GenBank: EF059877], NCTC 9844 [GenBank: EF059878], NCTC 9846 [GenBank: EF059879], z1084 [GenBank: AY803192], z759 [GenBank: AY752939], z858 [GenBank: AY752936], z954 [GenBank: AY752938], zES11 [GenBank: AY803187], zES4 [GenBank: AY803186], zESVO7 [GenBank: AY803189].

## Authors' contributions

CI performed the biochemical profiling and collated the data. AL and RS analyzed the 16S rRNA gene sequences. NM and SF performed the statistical analysis of the phenotypic data. EB and JM performed the Ribotyping and analysis. IC is responsible for the f-AFLP analysis at the BCCM/LMG Bacteria Collection, where the AFLP analyses were performed. HJ managed the project and edited the manuscript. All authors contributed to the text and read and approved the final manuscript.

## Supplementary Material

Additional file 1 Description of *E. sakazakii *biogroups. Click here for file
