# Supplementary material for: The taxonomy of Enterobacter sakazakii: proposal of a new genus Cronobacter gen. nov. and descriptions of Cronobacter sakazakii comb. nov. Cronobacter sakazakii subsp. sakazakii, comb. nov., Cronobacter sakazakii subsp. malonaticus subsp. nov., Cronobacter turicensis sp. nov., Cronobacter muytjensii sp. nov., Cronobacter dublinensis sp. nov. and Cronobacter genomospecies 1
Source: BMC Evol Biol. 2007 Apr 17;7:64. doi: 10.1186/1471-2148-7-64 (PMC1868726; doi:10.1186/1471-2148-7-64)
Supplement: Additional file 1 — Description of E. sakazakii biogroups. [file 1471-2148-7-64-S1.doc]

## Additional file 1. Description of *E. sakazakii* biogroups.

| Biogroup | Phenotype | | | | | | | | | | |
| --- | --- | --- | --- | --- | --- | --- | --- | --- | --- | --- | --- |
|  | VP | MR | Nit | Orn | Mot | Ino | Dul | Ind | Malo | Gas | AMG |
| 1 | + | - | + | + | + | + | - | - | - | + | + |
| 2 | + | - | + | + | + | - | - | - | - | + | + |
| 3 | + | - | + | + | - | + | - | - | - | + | + |
| 4 | + | - | + | - | + | + | - | - | - | + | + |
| 5 | + | - | + | + | + | + | - | - | + | + | + |
| 6 | + | - | + | + | + | + | - | + | - | + | + |
| 7 | + | - | + | + | + | + | - | - | - | - | + |
| 8 | + | - | - | + | + | + | - | - | - | + | + |
| 9 | + | - | + | + | + | - | - | - | + | + | + |
| 10 | + | - | + | + | + | - | - | + | - | + | + |
| 11 | + | - | + | + | + | - | + | - | - | + | + |
| 12 | + | - | + | + | + | + | - | + | + | + | + |
| 13 | - | + | + | + | + | + | - | - | - | + | + |
| 14 | + | - | + | - | + | - | - | - | + | + | + |
| 15 | + | - | + | + | + | + | + | + | + | + | - |
| 16 | + | - | + | + | + | + | + | - | + | + | + |

VP, Voges-Proskauer; MR, methyl red; Nit, nitrate reduction; Orn, ornithine decarboxylation; Mot, motility at 37°C; Ino, acid production from inositol; Dul, acid production from dulcitol; Ind, indole production; Malo, malonate utilization; Gas, gas production from glucose; AMG, acid production for methyl-α-D-glucoside.
